# Sentry Bioconvertible Inferior Vena Cava Filter: Study of Stages of Incorporation in an Experimental Ovine Model

**DOI:** 10.1155/2018/6981505

**Published:** 2018-07-19

**Authors:** Peter A. Gaines, Frank D. Kolodgie, Gordon Crowley, Steven Horan, Megan MacDonagh, Emily McLucas, David Rosenthal, Ashley Strong, Michael Sweet, Deepal K. Panchal

**Affiliations:** ^1^Hallam University, Sheffield S1 1WB, UK; ^2^Cardiovascular Pathology, CVPath Institute, Gaithersburg, Maryland, USA; ^3^Novate Medical Ltd, Galway, Ireland; ^4^Atlanta Vascular Specialists, Atlanta, Georgia, USA; ^5^Translational Testing and Training Laboratories (T3 Labs), Atlanta, Georgia, USA

## Abstract

The Sentry inferior vena cava (IVC) filter is designed to provide temporary protection from pulmonary embolism (PE) and then bioconvert to become incorporated in the vessel wall, leaving a patent IVC lumen.* Objective.* To evaluate the performance and stages of incorporation of the Sentry IVC filter in an ovine model.* Methods.* Twenty-four bioconvertible devices and 1 control retrievable filter were implanted in the infrarenal IVC of 25 sheep, with extensive daily monitoring and intensive imaging. Vessels and devices were analyzed at early (≤98 days,* n* = 10) and late (180 ± 30 days,* n* = 14 study devices, 1 control) termination and necropsy time-points.* Results.* Deployment success was 100% with all devices confirmed in filtering configuration, there were no filter-related complications, and bioconversion was 100% at termination with vessels widely patent. By 98 days for all early-incorporation analysis animals, the stabilizing cylindrical part of the Sentry frame was incorporated in the vessel wall, and the filter arms were retracted. By 180 days for all late-incorporation analysis animals, the filter arms as well as frames were stably incorporated.* Conclusions.* Through 180 days, there were no filter-related complications, and the study devices were all bioconverted and stably incorporated, leaving all IVCs patent.

## 1. Introduction

Pulmonary embolism (PE) leads to the hospitalization or death of approximately 225,000 Americans, 30,000 Canadians, and 300,000 Europeans per year, with the incidence having increased during the past decade [1, 2]. In the United States, annual estimates of the nonfatal occurrence of PE range from 400,000 to 630,000 [3], and it is the most common preventable cause of hospital-related death [4, 5]. Risk factors for PE include a history of deep vein thrombosis (DVT), major hospitalization and recent surgical procedures, traumatic injury, and prolonged inactivity or immobility [6]. The established primary treatment for venous thromboembolic (VTE) disease including PE and DVT is pharmacologic management with anticoagulant agents. However, in many patients anticoagulation is contraindicated during periods of high PE risk, and inferior vena cava (IVC) filters are recommended for these situations [3, 7–9].

In response to complications such as IVC thrombosis and perforation that have been associated with the originally developed IVC filters intended for permanent placement, various optional or retrievable IVC filters have become available since 2003 to provide protection from PE during recognized periods of transient risk, after which they are meant to be removed [10]. In practice, however, these filters have not been routinely retrieved, and while remaining indwelling, they have been associated with a time-dependent increase in retrievable-filter-specific complications including device tilting, fracture, migration, embolization, thrombosis, IVC perforation, surgery, and death [4, 11–15]. In April 2010, the US Food and Drug Administration (FDA) issued a safety communication (which was updated in May 2014) recommending that physicians “consider removing the filters as soon as protection from pulmonary embolism is no longer needed” [9, 16, 17]. According to a subsequently published FDA decision analysis, the benefit/risk profile begins to favor filter removal between 29 and 54 days after device implantation [16]. Nevertheless, even with a consequent increase of education and patient-tracking initiatives, as many as 65% to 80% of filters remain unretrieved [4, 12–15].

**Figure 1:**
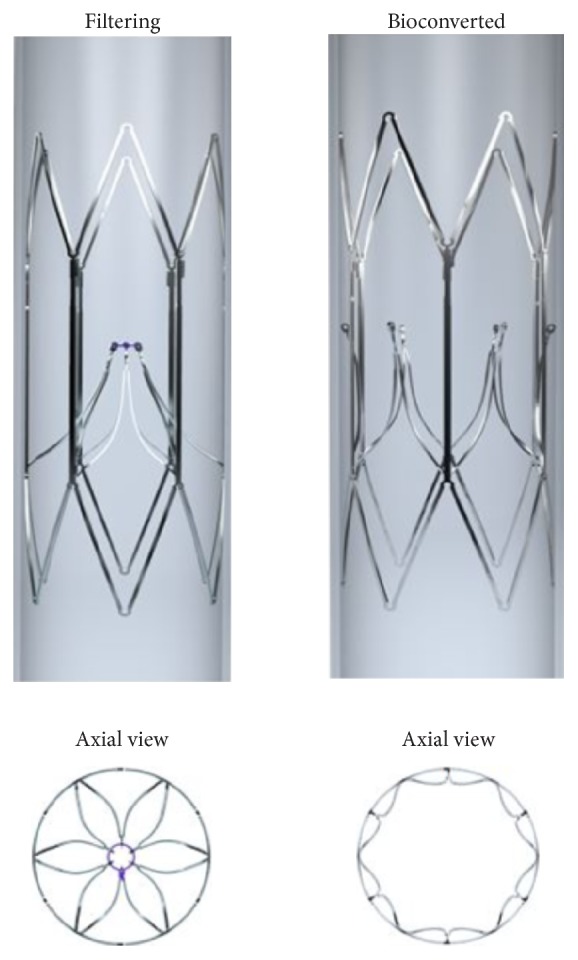
Sentry IVC filter in filtering (left) and bioconverted (right) configurations.

The Sentry IVC filter (Novate Medical Ltd, Galway, Ireland) is designed to provide temporary protection from PE during transient high-risk periods and then to bioconvert, thus avoiding the need for a second (retrieval) intervention while leaving a patent IVC lumen. Within a stabilizing cylindrical nitinol frame, the Sentry filter cone is formed by 6 pairs of arms held together at an apex in the center of the lumen by a bioabsorbable filament (Figure 1). Bioconversion is achieved with the hydrolysis of the filament material allowing the filter arms to retract to the IVC wall, where they are incorporated along with the cylindrical frame, leaving the vessel lumen patent. The endothelialization of the device after the bioconversion and retraction of the arms is expected to limit the likelihood of late filter-related complications. The objective of this preclinical study was to evaluate the technical performance, stages of incorporation, and any device-related complications associated with the Sentry IVC filter in an ovine model with intensive* in vivo* and* ex vivo* imaging analysis.

## 2. Materials and Methods

### 2.1. Device Description

The Sentry IVC filter consists of a laser-cut one-piece nitinol device, comprising a cylindrical frame and a filter cone formed by 6 pairs of arms that are held together in the center of the IVC lumen by means of a bioabsorbable filament. The filament is composed of poly-*p*-dioxanone (PPDO), a synthetic polymer that degrades naturally* in vivo* via hydrolysis and has been used in biodegradable devices (sutures, for example) since the 1970s [18]. The nitinol frame is designed to concentrically and longitudinally distribute radial force in order to decrease device tilting, migration, and perforation. Upon deployment, the cylindrical frame expands to appose the IVC wall, commencing incorporation and neointimal healing. The bioabsorbable filament is designed to hydrolyze so that the arms are released from the filtering cone and retract to the IVC wall into a nonfiltering configuration. This design allows temporary protection against PE, and following this bioconversion leaves the IVC lumen patent. The Sentry is indicated for use in IVCs with diameters between 16 and 28 mm. The filter comes preloaded in a loading tool, which is attached to a custom introducer sheath for deployment. It is suitable for a femoral or jugular approach. The filter is advanced through the introducer sheath using a pusher, which is supplied with the device. Once the device is at the intended location, the pusher is held stationary and the introducer sheath is retracted to execute deployment.

### 2.2. Animal Model

Sheep are known to be suitable anatomically and physiologically for the evaluation of cardiovascular devices, including IVC filters, and have been used extensively in preclinical studies [19–21]. The coagulation system of sheep has been reported to be more similar to that of humans than the coagulation systems of pigs or dogs, although a tendency toward hypercoagulability has been reported with the ovine model, based on hematological differences related to platelet count and the size of red blood cells [21]. Suffolk cross sheep were selected for this study, because previous experience with this breed has shown that IVC diameter tends to be larger than in other breeds and closer to the vessel diameter indicated for the Sentry IVC filter (16 to 28 mm), thus ensuring more representative clinical performance evaluations. The sheep used in this study were all castrated males (wethers) between 1 and 2 years of age and weighing between 54.0 and 79.8 kg at the time of study device implant.

### 2.3. Study Design

The objectives of this preclinical study were to assess technical performance with the Sentry IVC filter in terms of deployment, positioning in the IVC, confirmation of intended filtering configuration, and incorporation following device bioconversion; to evaluate the early* in vivo* response to the cylindrical filter frame and the late incorporation of the device into the IVC wall after 6 months* in vivo*; and to assess IVC filter-related complications through 6 months. The study was performed at Transitional Testing and Training Laboratories (T3 Labs, formerly St. Joseph's Translational Research Institute), Atlanta, GA, USA, in accordance with the requirements of 21 CFR Part 58 (Good Laboratory Practice). Animal care (including purchase, transport, quarantine, and acclimation; health and medication monitoring; use of sedatives, analgesics, and anesthesia; and euthanasia) was conducted in accordance with US Department of Agriculture guidelines through the Animal Welfare Act (9 CFR Parts 1, 2, and 3) and guidelines of the Institute of Laboratory Animal Resources of the National Academy of Sciences and the Association for Assessment and Accreditation of Laboratory Animal Care International.

**Figure 2:**
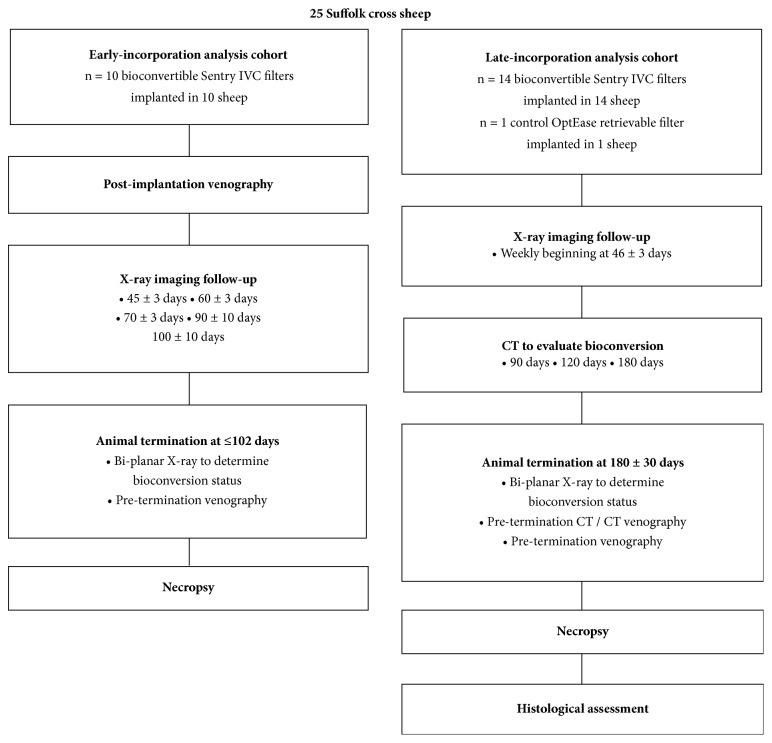
Study design.

The study design is summarized in Figure 2. A total of 24 bioconvertible study devices and 1 control retrievable filter were implanted in the infrarenal IVC of 25 sheep, with extensive daily monitoring and intensive imaging and posttermination necropsy. The devices and animals were treated and evaluated in two distinct cohorts: (1) early-incorporation analysis cohort, for assessment of device performance, bioconversion, and incorporation of the cylindrical frame, with termination and necropsy performed ≤98 days after implantation (n = 10 study devices); (2) late-incorporation analysis cohort, for assessment of device incorporation (cylindrical frame and retracted filter arms), with termination and necropsy planned for 180 ± 30 days after implantation (n = 14 study devices and 1 control device). All 24 Sentry devices implanted in the two study cohorts were identical in terms of frame and filter arm design and the material of the bioabsorbable filament.

The one control device implanted in the late-incorporation analysis cohort was an OptEase Retrievable Vena Cava Filter (Cordis, Milpitas, CA, USA), a commercially available device.

### 2.4. Treatment Procedure

Prior to enrollment, all sheep were quarantined and acclimated. All animals were given sedatives, analgesics, and anesthesia medications in accordance with the facility procedures. Heparin was administered as needed to maintain an activated clotting time (ACT) of >300 seconds during the procedure.

Prior to implantation, percutaneous access was gained and an introducer sheath was placed in either the right or left femoral vein. Biplanar venography (anteroposterior and lateral) was performed under respiratory apnea to determine IVC diameter and assess vessel geometry to ensure suitability for enrollment. The IVC diameter was determined by calculating the average of the 2 measurements. A 5 Fr pigtail catheter (Cook Medical, Bloomington, IN, USA) was used for calibration purposes. Once the sheep met the inclusion criteria, in terms of IVC geometry and diameter, the Sentry introducer sheath was placed. One Sentry IVC filter was deployed in the IVC of each animal in accordance with the device instructions for use (IFU). Every effort was made to only enroll animals that were within the indicated vessel diameter range of 16 to 28 mm. Following implantation, all cannulation sites were closed and the animals were recovered.

### 2.5. In-Life Monitoring

Animals were monitored daily to check on feeding patterns, mental attitude, and physical responsiveness. Temperature, pulse, and respiration (TPR) checks and subjective objective assessment plans (SOAP) were performed weekly. The SOAP included an assessment of sheep movement, feeding and stool, and mucous membranes.

In the early-incorporation analysis cohort, X-ray follow-up was performed at 45 (±3), 50 (±3), 60 (±3), 70 (±3), 90 (±10), and 100 (±10) days after implantation. In the late-incorporation analysis cohort, weekly X-rays were performed starting on day 46 (±3) after implantation. A portable X-ray machine (MinXray HF 100, Northbrook, IL, USA) was used with the animals humanely restrained in a standing position, and no sedation was required. In the late-incorporation analysis cohort, computed tomography (CT) was used to evaluate bioconversion at 90, 120, and 180 days. At 180 days, CT angiography was also performed. Prior to termination for all animals in both cohorts, biplanar X-rays were obtained to determine the filter bioconversion status.

### 2.6. Termination

Prior to termination, all animals were sedated per standard facility procedures and an introducer sheath was placed in the femoral vein. Venography was performed on all animals. CT and CT venography were performed on animals in the late-incorporation analysis cohort. Heparin was administered as needed to maintain ACT at >300 seconds during the termination procedure. Following the in-life imaging, all animals were euthanized under deep inhalant anesthesia, using 20 to 40 mEq potassium chloride in accordance with the facility's procedures, and were then sent for necropsy.

### 2.7. Necropsy Assessment

All scheduled necropsy was performed by a board-certified pathologist. The abdominal and thoracic cavities were opened and a macroscopic evaluation of the implant site was performed. The IVC was perfusion fixed with 10% neutral buffered formalin (NBF). The lungs were examined for gross evidence of pulmonary infarction, and samples from each lobe were obtained and immersion fixed in 10% NBF.

Representative sections of the following organs were immersion fixed in 10% NBF and retained: spleen, heart, liver, kidneys, brain, and local lymph nodes. Additional organs were collected as deemed appropriate. All tissues were imaged using standard digital photography. In addition, a laparoscope was used to image the internal lumen of the IVC section containing the device.

### 2.8. Histological Assessment

Histological assessment was performed on all animals in the late-incorporation analysis cohort (animals euthanized at 180 ± 30 days postimplantation) by CVPath Institute (Gaithersburg, MD, USA). All IVC sections containing IVC filters were imaged using high-contrast digital-based radiographs (Faxitron X-Ray Corporation, Model LX-60, Lincolnshire, IL, USA). The radiographs were used during sectioning and were also examined for potential fractures.

All IVC sections containing IVC filters were processed in a graded series of alcohols and xylenes. The IVCs were embedded in resin and sections were prepared with a microtome or ground using EXAKT (Oklahoma City, OK, USA) Linear Grinding technology. Ground sections were stained with toluidine blue/basic fuchsin, and sections prepared with a microtome were stained with hematoxylin and eosin (H&E) or Movat pentachrome.

Morphometric measurements were obtained for cross-sectional areas, relative filter arm expansion, neointimal thickness, and percent stenosis. Tissue incorporation along the length of the IVC filters was evaluated, and the neointimal coverage and apposition of the filter frame, arms, and tips were assessed by semiquantitative analysis. Filter arms and tips were also analyzed for the presence of residual PPDO filament. The lungs were dehydrated in a graded series of alcohols and xylene. The samples were then embedded in paraffin, sectioned on a rotary microtome, and stained with H&E.

### 2.9. Data and Statistical Analysis

Basic statistical analysis, including the calculation of mean values and standard deviations, was performed on relevant data tables using a registered copy of Microsoft Excel.

## 3. Results

### 3.1. Filter Deployment and Initial Follow-Up

**Figure 3:**
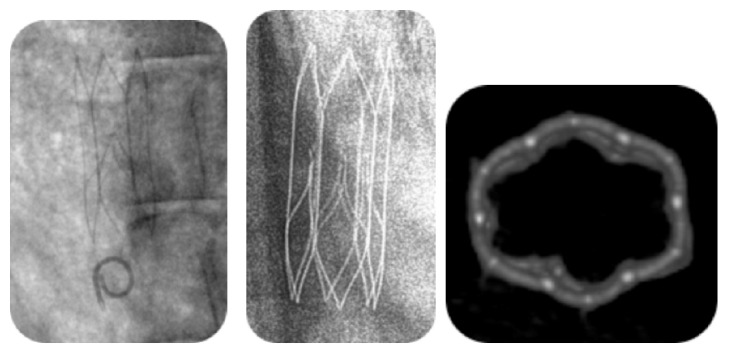
Representative imaging for an animal from the late-incorporation analysis cohort. Left: venogram of the Sentry IVC filter on the day of implantation. Middle: X-ray of the bioconverted filter. Right: CT reconstruction showing bioconversion of the Sentry IVC filter.

Since the IVC tends to be oval [22], the IVCs of all sheep were measured in both anteroposterior and lateral views, and the IVC diameter was determined by calculating the average of the 2 measurements. Overall for the two cohorts the mean IVC diameter was 17.3 mm (range 14.2 to 22.2 mm). All 25 devices were accurately deployed as intended and were placed below the renal veins in all cases. Upon implantation, the alignment of the filters with the IVC wall was confirmed, with the filter apex being visible and well centered (Figure 3).

After implantation, the animals were monitored at the animal trial facility. One animal from the early-incorporation analysis cohort died prematurely 8 days after implantation. The gross necropsy assessment of the IVC, lungs, bladder, kidneys, liver, spleen, heart, brain, and lymph nodes of this animal reported a probable cause of death as septic emboli related to lung abscess. The detailed histologic evaluation on selected lung and myocardial tissue concluded that the suspected cause of death was pulmonary infection leading to a hypercoagulable state. The premature death was not attributed to the Sentry IVC filter.

### 3.2. Early-Incorporation Analysis Cohort

The remaining 9 sheep from this cohort were terminated ≤98 days after implantation—1 at 51 days after implantation, 1 at 89 days, 4 at 97 days, and 3 at 98 days. There was no evidence of any device-related complications, and the IVCs were patent in all animals.

**Figure 4:**
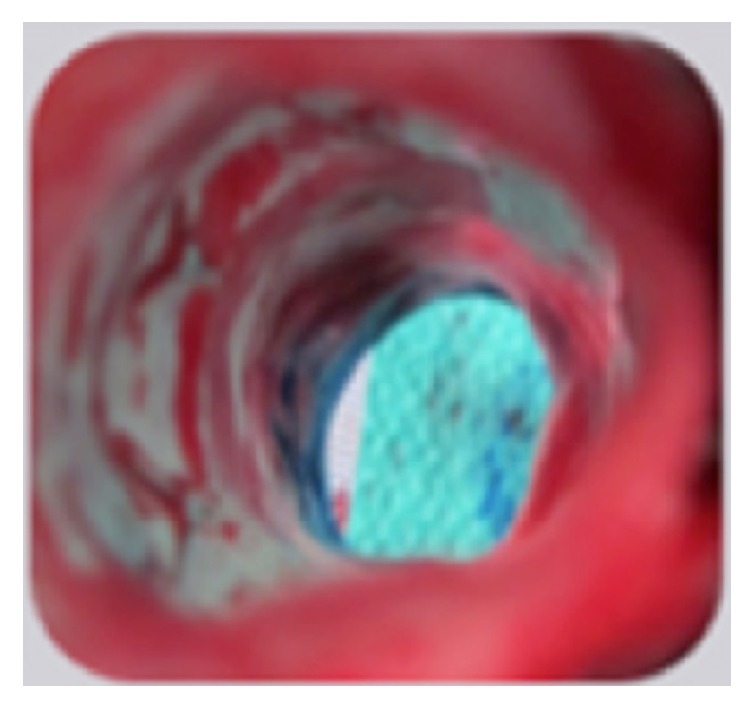
IVC from an animal from the early-incorporation analysis cohort terminated at 97 days. Laparoscopic imaging at necropsy of IVC sections containing the Sentry IVC filter, showing the unobstructed patent lumen and the nearly complete incorporation of the filter frame.

During necropsy, all IVCs were assessed macroscopically to determine the level of patency within the IVC and evaluate the progression of incorporation ≤98 days after implantation. In each case, a laparoscope was used to axially image the IVC section containing the Sentry IVC filter, and the IVC section containing the filter was cut longitudinally to facilitate visual assessment of incorporation. The IVCs were patent in all animals, with no caval thrombosis, the cylindrical frames of the filters were well incorporated, and the filter arms had retracted to the vessel wall, with incorporation commenced but not yet complete.  Figure 4 shows the unobstructed patent lumen and the nearly complete incorporation of the filter frame (which is almost invisible) from an animal that was terminated 97 days after implantation.

### 3.3. Late-Incorporation Analysis Cohort

The sheep in this cohort underwent CT follow-up at 104-112 days (falling within the 120-day time window), at 139-147 days (falling within the 150-day window), and at 174-189 days (the 180-day window). Beginning with the 120-day CT imaging and continuing with the 150-day and 180-day imaging, all of the 14 study devices were in nonfiltering configuration. The 180-day CT venography demonstrated IVC lumen patency in all of the study animals.

The animals in this cohort were terminated between 180 and 209 days after implantation. Pretermination venography and CT/ CT venography of the control device, the OptEase Retrievable Vena Cava Filter (the animal was terminated on day 183 after implantation), identified no IVC filter-related complications. The pretermination imaging showed that all Sentry IVC filters were bioconverted (Figure 3) and that the IVCs were patent with excellent flow. There was no evidence of any device-related complications (no device tilting, fracture, migration, perforation, or IVC occlusion or thrombus) and no evidence of IVC stenosis.

After termination of the animals and prior to explant of the IVC sections with the implanted filters, digital photographs were obtained to evaluate the impact of the filter on the surrounding tissue. Based on these images, it was determined that the Sentry IVC filters were well tolerated by the sheep, whereas there was significant transmural incorporation of the control OptEase device.

**Figure 5:**
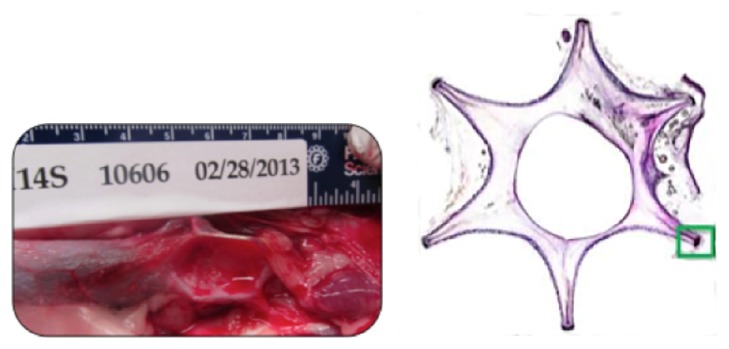
Imaging of the IVC segments with the control OptEase device (terminated at 183 days). Left: preexplant digital photograph of IVC sections with the implanted control OptEase Retrievable Vena Cava Filter. Right: a transverse section stained with toluidine blue/basic fuchsin, demonstrating incorporation of the device into the wall of the IVC.

In laparoscopic imaging performed* ex vivo* to evaluate patency of the IVC with the control OptEase device, the cylindrical portions of the frame were found to be mostly incorporated into the vessel wall. The filtering apices of this control device were within the IVC lumen, and the caudal apex was associated with adherent fibrotic tissue. Overall, the OptEase device produced notable overstretch of the IVC wall, producing a star-shaped morphology, with moderate stenosis and thickness in the central lumen (Figure 5).

**Figure 6:**
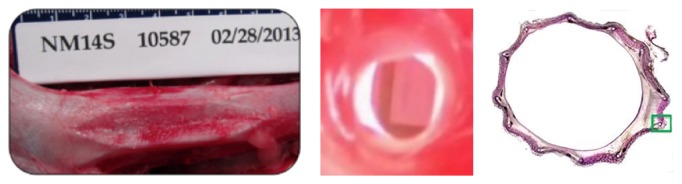
Representative images of IVC sections with the Sentry IVC filter (terminated at 184 days). Left: preexplant digital photograph of IVC sections with the implanted Sentry device. Middle: laparoscopic image of IVC filter within the IVC, demonstrating lumen patency. Right: transverse section stained with toluidine blue/basic fuchsin, demonstrating incorporation into the wall of the IVC. The green box indicates mild neointimal proliferation.

The* ex vivo* laparoscopic examination of IVC segments with the Sentry device was for evidence of perforation or hemorrhage, for the incorporation of the filter frame, for the apposition of the filter arm tips to the IVC wall, and for biological material at the filter arm tips. There was no evidence of perforation or hemorrhage in any of the animals, and the filter frames were fully incorporated in all cases (Figure 6).

**Figure 7:**
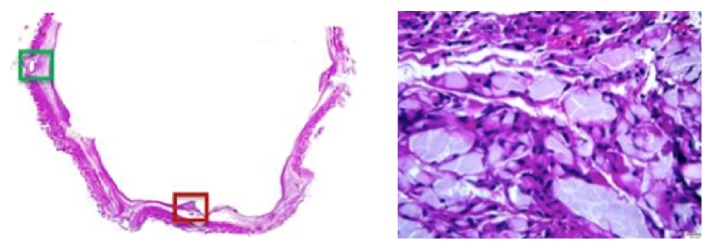
Representative high-power images from the histological analysis of the integration of the Sentry filter arms into the wall of the IVC. Left: section cut through the tips of the filter arms, stained with H&E. Right: view of residual PPDO filament material, surrounding chronic inflammatory cells adjacent to the fully incorporated tip of a filter arm (dark red box in the image at left).

Histological analysis demonstrated that the cylindrical portions of the Sentry IVC filter frames were covered by neointima, with the exception of some uncovered portions, which were generally near tributary vessels. The devices were stably incorporated into the wall of the IVC, with minimal inflammation and good endothelialization. The retracted filter arms demonstrated good integration into the wall of the IVC. Residual filament was visible at the eyelets in some devices and there was mild inflammation consisting of macrophages near the filament.  Figure 7 shows a representative section through the tips of the filter arms, stained with H&E, and a high-power magnification image of residual PPDO filament, surrounded by chronic inflammatory cells. The residual filament was located adjacent to the fully incorporated tip of a filter arm. None of the organ sections including the lung sections showed any emboli or evidence of filament.

## 4. Discussion

Employing extensive clinical and imaging-intense follow-up (before and after animal termination) in an ovine model, this experimental study provides support for the utility of the Sentry IVC filter and the realization of the device concept of protecting against PE during periods of transient risk and then leaving a patent vessel lumen without the need for secondary interventions to perform retrieval. In all 24 animals that received study devices, deployment and positioning in the filtering configuration in the center of the IVC lumen were successful, and pre- and posttermination examinations confirmed that all filters had bioconverted as intended, leaving the IVC lumens patent. For the animals with termination at ≤98 days, final in-life venography and necropsy showed that the stabilizing cylindrical part of the Sentry frame was incorporated in the vessel wall, and the filter arms were retracted. For the animals with termination at 180 ± 30 days, the final in-life imaging, necropsy, and histopathology confirmed that the filter arms as well as frames were stably incorporated and that the IVC lumens were unobstructed and patent. Through 180 days, there were no filter-related complications.

Retrievable IVC filters were developed to meet the need for transient protection from PE in patients with temporary contraindications to anticoagulants [6]. Considerable research supports the premise that the period of highest risk for PE is relatively early for individuals in such situations. In one study in a group of trauma patients, the average time from injury to PE was determined to be 7.9 days [23], and other studies have found that the majority of trauma-related PE occurred less than 30 days after the index event [24, 25]. In studies of postoperative PE, the mean time from surgery to PE was 3 to 20 days [8, 26, 27]. The majority of in-patient PE events after orthopedic surgery occur within 35 days after the procedure [28, 29]. When the transient period of highest PE risk has passed, retrieval can reduce the incidence of long-term complications associated with indwelling IVC filters—such as caval thrombosis, DVT, perforation, and filter fracture [30–33].

However, the increasing use of retrievable IVC filters has brought some important safety and practical issues into focus. The need to develop devices that will effectively trap emboli but then be readily retrievable has resulted in relatively unstable conical-type designs that demonstrate less secure implantation than the permanent filters [34]. These design compromises have led to complications such as tilting, migration, and fracture [30, 34]. In practice, retrieval rates remain low, despite FDA engagement. A retrospective review performed by Angel et al. reported an average retrieval rate of 34% in 6834 patients (range 12% to 45%) across 37 clinical studies [4]. One study demonstrated that even with a dedicated filter registry only 59% of filters could be successfully removed [35].

The* in vivo* studies demonstrate that the cylindrical design of the nitinol frame of the Sentry IVC filter provides stability in apposition to the wall of the IVC that is not provided by retrievable filters, thus reducing complications such as tilting and migration. Incorporation of the cylindrical portion of the frame is advanced by 100 days after implantation, while the filter arms are stably incorporated into the vessel wall by 180 days after implantation. The laparoscopic and histological assessment provides corroborating support regarding the full degree of filter endothelialization and vessel patency with no evidence of IVC stenosis, thus demonstrating the restoration of normal tissue function by 180 days.

IVC filters are designed to capture thrombus. Studies by Singer et al. employing computational fluid dynamics (CFD) indicate that the flow disturbances initiated by IVC filters can cause regions of stagnation, which can potentially contribute to thrombosis and caval occlusion [36, 37]. In the PREPIC (Prévention du Risque d'Embolie Pulmonaire par Interruption Cave) randomized trial evaluating the long-term outcomes of patients with proximal DVT, investigators concluded that the 8-year incidence of recurrent DVT was significantly higher in the group of 200 patients implanted with permanent IVC filters than in the 200 patients who did not receive filters [33]. The direct cause of the increased rate of DVT is not clear, but it is conceivable that long-term filter-induced flow disturbances had a contributory effect [36, 37].

The concept of “leaving nothing behind” after percutaneous interventions, which has emerged in recent years with the development of biodegradable stents [38], has an appeal for the prevention of PE, considering the transient nature of the need for protection in the majority of patients receiving IVC filters and the high complication rates when timely device retrieval does not occur [16]. The PREPIC trial and the CFD analyses performed by Singer et al. highlight the benefits of restoring normal blood flow in the IVC after the risk of PE has passed. The Sentry IVC filter design allows timely disassembly of the filtering mechanism, leaving nothing behind in the lumen of the IVC to obstruct blood flow. The body's normal healing processes, combined with the nitinol frame design of the Sentry device, support the restoration of normal tissue function within the IVC. The Sentry IVC filter thereby offers the benefits of a retrievable IVC filter, without the burden and cost of a second procedure, the design compromises required to facilitate retrieval, or the risks of nonretrieval.

This preclinical study employed extensive imaging-intense follow-up—involving scheduled X-ray, CT, CT venography, and venography. Comparatively, it was found that X-ray was effective for assessing device filtering configuration and bioconversion status with filter arm separation from the filament cone, that CT was useful for assessing the retraction status of the filter arms in terms of their separation from the cone and from each other, and that CT venography could be utilized to identify thrombus and (in combination with plain CT) to determine the adjacency of the filter arm tips to the IVC wall. Venography was useful for identifying thrombus and patency of and blood flow in the IVC lumen, but it was of limited use for determining the positions of filter arm tips.

The limitations of this study include the fact that there is no definitive evidence to support the assumption that the sheep IVC represents a valid model of the human IVC, and all findings (positive and negative) in the model can only serve as inferences with respect to the performance of the study device in humans. However, the model justification process for the study carefully considered the comparability to humans of the sheep model relative to other models such as dogs and pigs, in terms of features including overall size and IVC diameter (which was optimized by selection of the Suffolk cross breed), respiration rate, cardiac output, temperature, and blood characteristics. The tendency toward hypercoagulability in the sheep model was noted, and the investigators also noted that the sheep anatomy provided a greater challenge than in humans for deployment, due to extreme bending of the introducer sheath imposed by the groin geometry. The rate of hydrolysis of the PPDO filament is directly dependent on temperature, and human blood temperatures are lower than ovine blood temperatures [39]. Therefore, bioconversion times in an ovine model are not representative of device opening times* in vitro,* and while X-rays were performed during the animal studies to understand the process of bioconversion, the data collected were not used to establish the time to opening of the device. The sample size of 24 animals implanted with study devices, while small in comparison with some human studies, was comparable to or greater than in preclinical studies of other (retrievable) IVC filters. The 180-day duration of the study allowed demonstration of component endothelialization and device stability [19, 20, 40, 41].

## 5. Conclusions

With extensive imaging-intense follow-up, this preclinical study in a sheep model provided important information about the technical performance, bioconversion, and stages of incorporation of the Sentry IVC filter. The preclinical study supports the premise that the Sentry IVC filter design allows timely disassembly of the filtering mechanism, leaving nothing behind in the lumen of the IVC to obstruct blood flow. A prospective multicenter pivotal investigational device exemption trial of the Sentry IVC filter is ongoing (NCT01975090).

## Data Availability

The dataset analyzed for this study is available from the corresponding author upon reasonable request.
